# Localized Deformation and Fracture Behaviors in InP Single Crystals by Indentation

**DOI:** 10.3390/mi9120611

**Published:** 2018-11-22

**Authors:** Yi-Jui Chiu, Sheng-Rui Jian, Ti-Ju Liu, Phuoc Huu Le, Jenh-Yih Juang

**Affiliations:** 1 School of Mechanical and Automotive Engineering, Xiamen University of Technology, No.600 Ligong Road, Jimei District, Xiamen 361024, China; chiuyijui@xmut.edu.cn; 2 Department of Materials Science and Engineering, I-Shou University, Kaohsiung 840, Taiwan; diru@isu.edu.tw; 3 Department of Physics and Biophysics, Faculty of Basic Sciences, Can Tho University of Medicine and Pharmacy, 179 Nguyen Van Cu Street, Can Tho 94000, Vietnam; 4 Department of Electrophysics, National Chiao Tung University, Hsinchu 300, Taiwan

**Keywords:** InP(100) single crystal, Pop-in, nanoindentation, transmission electron microscopy, fracture toughness

## Abstract

The indentation-induced deformation mechanisms in InP(100) single crystals were investigated by using nanoindentation and cross-sectional transmission electron microscopy (XTEM) techniques. The results indicated that there were multiple “pop-in” events randomly distributed in the loading curves, which were conceived to arise primarily from the dislocation nucleation and propagation activities. An energetic estimation on the number of nanoindentation-induced dislocations associated with pop-in effects is discussed. Furthermore, the fracture patterns were performed by Vickers indentation. The fracture toughness and the fracture energy of InP(100) single crystals were calculated to be around 1.2 MPa·m^1/2^ and 14.1 J/m^2^, respectively.

## 1. Introduction

Nowadays, nanoindentation is extensively used to characterize the mechanical properties (such as hardness and elastic modulus) and elastic or plastic deformation behaviors of various nanoscale materials [1,2,3,4,5,6] and thin films [7,8,9,10,11,12]. In general, from the nanoindentation responses manifested in the load-displacement (*P*-*h*) curves, one can obtain the primary mechanical characteristics of the materials being measured. For instance, the onset of plastic deformation behaviors in crystalline materials is often characterized by sudden bursts of displacements at a nearly constant indentation load in the *P*-*h* curves. These phenomena, known as “pop-in,” have been ubiquitously observed and often considered to be a result of dislocation activity during the nanoindentation process [13,14]. Lorenz et al. [15] proposed that the pop-in event is originated from homogeneous dislocation nucleation beneath the indenter tip. This scenario is reasonably in line with the low probability of encountering the pre-existing dislocations. Furthermore, previous cross-sectional transmission electron microscopy (XTEM) observations [14,16,17] evidenced the intimate correlations between pop-in events and dislocation activities in many nanoindentation studies [14,15,16,17].

Owing to its high electron velocity and the direct bandgap (~1.35 eV), zincblende-structured indium phosphide (InP) has been regarded as one of the most important III–V semiconductors. Currently, applications based on InP have been realized in a wide variety of electronic and photonic devices and systems, such as high-power and high-frequency electronics, solar cells, photodiodes, photodetectors, light-emitting diodes (LEDs), field effect transistors (FETs), and micro-electro-mechanical systems (MEMS) [18,19,20]. From an application point of view, in addition to its optoelectronic properties, a full understanding of the mechanical properties of InP is equally essential in order to widen its applications and manipulate the performance of devices.

For InP(100) single crystals, however, two different types of *P*-*h* curves obtained from nanoindentation studies were reported, namely single-discontinuity [21] and multi-discontinuities [22]. Such behavior remains intangible because the onset of nanoscale plasticity can be strongly influenced by various factors, such as indenter tip radius, temperature, and crystal plane [23,24]. Therefore, in order to understand nanoindentation-induced pop-in mechanisms and clarify the outstanding issues of nanoscale plasticity in InP(100) single crystals, a combination of XTEM and selected area diffraction (SAD) analyses were carried out in this work. The number of nanoindentation-induced dislocation loops in InP(100) single crystals was estimated within the context of the classical dislocation theory [25]. Moreover, the Vickers-indentation induced fracture toughness and fracture energy of InP (100) single crystals were calculated and discussed in details.

## 2. Materials and Methods

**Figure 1:**
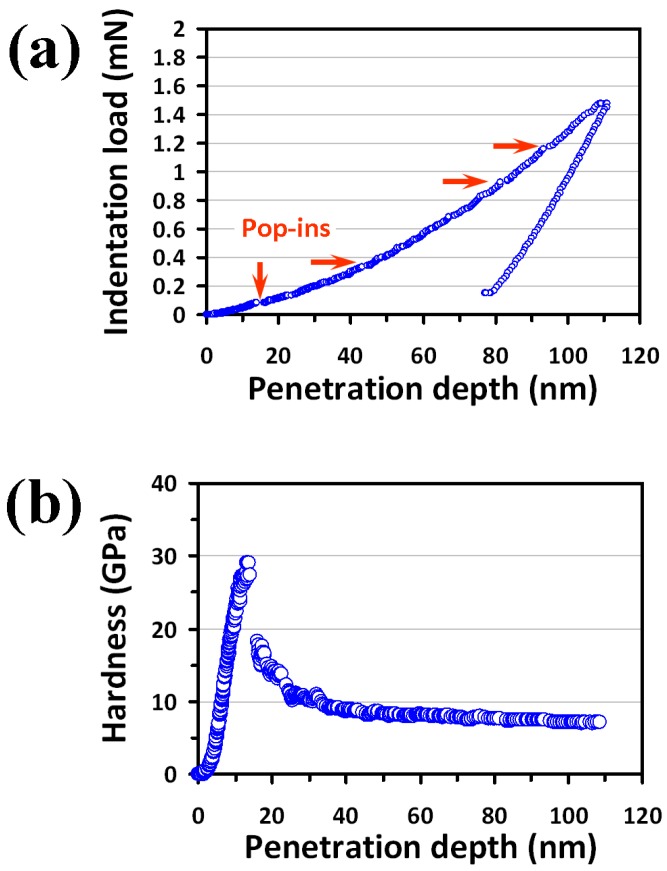

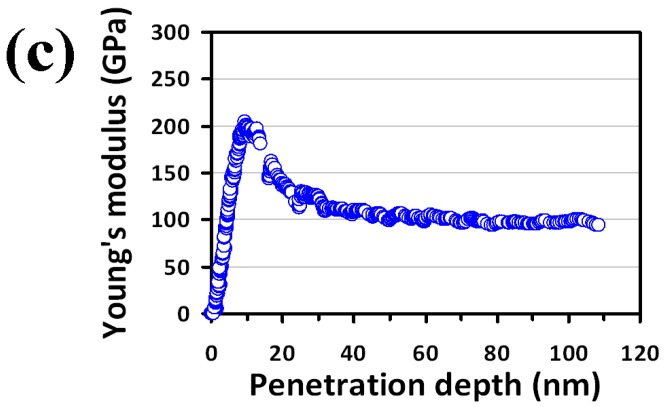
Nanoindentation results of single-crystal InP(100): (**a**) load-displacement curve showing the multiple “pop-ins” (arrows) during loading, (**b**) hardness-displacement curve and, (**c**) Young’s modulus-displacement curve.

The (100)-oriented single-crystal InP used in this work was purchased from Semiconductor Wafer Inc. (Hsinchu, Taiwan) The nanoindentation tests were performed using a Nanoindenter MTS NanoXP^®^ system (MTS Cooperation, Nano Instruments Innovation Center, Oak Ridge, TN, USA) with a diamond pyramid-shaped Berkovich indenter tip having a radius of curvature of ~50 nm. The mechanical properties of single-crystal InP(100) were obtained using the continuous stiffness measurements (CSM) technique, commonly practiced in the nanoindentation community [26]. Hardness and Young’s modulus of single-crystal InP(100) were obtained using Oliver and Pharr method [27], as shown in Figure 1.

The hardness of the measured material is defined as the applied indentation loading divided by the projected contact area, *H* = *P_m_*/*A_p_*, where *A_p_* is the projected contact area and *P_m_* is the maximum indentation load. For a perfect Berkovich indenter, the projected area is given by $A_{p}=24.56h_{c}^{2}$ with *h_c_* being the contact depth. In addition, the elastic modulus of the material can be calculated based on the relationship proposed by Sneddon [28]: $S=2\beta E_{r}\sqrt{A_{p}}/\sqrt{\pi }$. Here, *S* is the contact stiffness of the material and β is a geometric constant, with β = 1.00 for the Berkovich indenter. The reduced elastic modulus, *E_r_*, can be calculated from the following equation:

(1) $$\frac{1}{E_{r}}={\left(\frac{1-v^{2}}{E}\right)}_{d}+{\left(\frac{1-v^{2}}{E}\right)}_{InP}$$

with *v* and *E* being Poisson’s ratio and Young’s modulus, respectively. The subscripts “*d*” and “*InP*” indicate the properties of the indenter and material, respectively. For the diamond indenter tip, $E_{d}$ = 1141 GPa, $v_{d}$ = 0.07 [27], and $v_{InP}$ = 0.25 were assumed for single-crystal InP(100).

**Figure 2:**
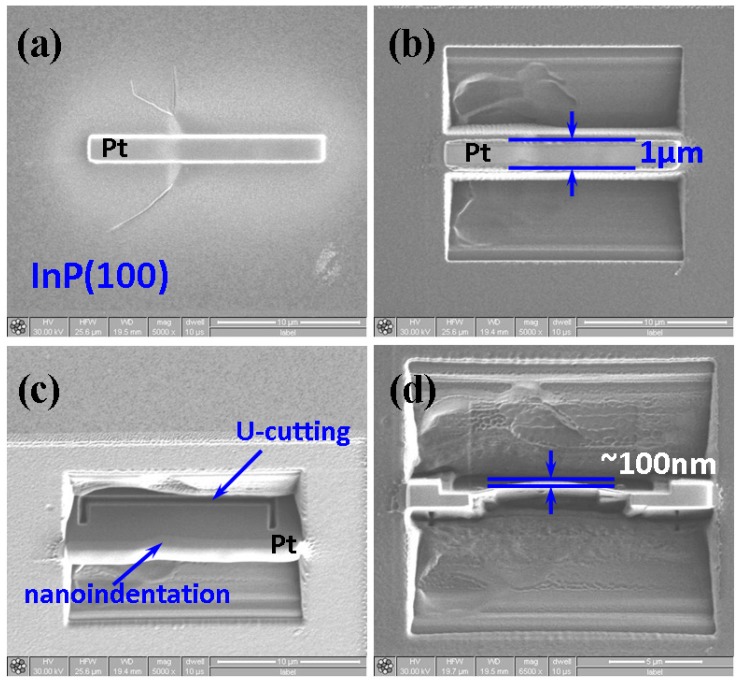
A typical procedure of focused ion beam (FIB) milling for single-crystal InP(100) is shown. Sample preparation starts with a line of nanoindentations. After depositing a protection layer of Pt (**a**), two big trenches are etched on either side of the indentation line by a high current ion beam (7~20 nA) (**b**) Further, the middle strip is thinned (**c**). An ion dose of 50 pA is used for final clearing steps and, finally thinned to a thickness of ~100 nm (**d**).

After being deformed by an indentation load of 150 mN, XTEM samples of InP(100) single crystals were prepared using a dual-beam focused ion beam (FIB) station (FEI Nova 220) with the lift-out technique. Pictorial illustrations of the FIB milling procedures are displayed in Figure 2. The sample was then picked up by a carbon membrane and placed on the TEM grid using a sharp glass tip under an optical microscopy (OM) outside the FIB station. The XTEM lamella was examined in a FEI TECNAI G^2^ TEM operating at 200 kV.

Vickers indentation testing was carried out in single-crystal InP(100) to characterize the cracking behavior with five indents at a loading of 1.96 N by using a hardness tester (Akashi MVK-H11, Kanagawa, Japan). All indentations were made in ambient air at room temperature with a relative humidity of about 55%. The cracking patterns were examined and analyzed with an optical microscope (OM).

## 3. Results

### 3.1. Nanoindentation Responses

A typical CSM *P*-*h* curve of single-crystal InP(100) reflecting the elastic behavior and plastic deformation during nanoindentation is shown in Figure 1a. The results clearly show that there are several pop-ins occurring at different loading stages, as indicated by the arrows located at different indentation loadings, which is consistent with a previous report [22]. Hardness and Young’s modulus versus penetration depth curves obtained from the CSM analyses for single-crystal InP(100) are displayed in Figure 1b,c, respectively. Both curves exhibit very similar depth-dependent trends, namely an initial quasi-linear increase to a maximum value within the first 10–15 nm, followed by a subsequent steep decrease in the 20–30-nm range, and finally reaching a constant value. It is interesting to note that the steep decrease after the first stage essentially coincides with where the first pop-in event is observed, indicating that a bursting activity of dislocation might have occurred.

For a uniform material, hardness and Young’s modulus do not change significantly with increasing penetration depth. The initial increase seen in Figure 1b,c is owing to the fact that the practical indenter tip is of a finite radius of shape point. This effectively sets the limit on the indentation depth that is necessary to obtain reliable hardness and Young’s modulus records of the measured material. As shown in Figure 1b,c, hardness and Young’s modulus reach a constant value at a similar moderate indentation depth. Thus, the values of hardness and Young’s modulus obtained at this stage can be regarded as intrinsic properties of single-crystal InP(100). In this report, both mechanical parameters were determined by taking the average values within a penetration depth ranging from 60 nm to100 nm.

Hardness and Young’s modulus of single-crystal InP(100) thus obtained are about 7.5 GPa and 101.8 GPa, respectively. These values are both substantially larger than those reported by Bradby et al. [21], where hardness and Young’s modulus for InP(100) were ~5.1 GPa and ~82 GPa, respectively. We note that in their experiments, a spherical indenter with a radius of ~4.2 μm and a load up to 50 mN were used, whereas in the present study a pyramid shape Berkovich indenter with a tip radius of ~40 nm (facing 65.3° from the vertical axis) and a typical load of less than 2 mN were used. It is reasonable to speculate that in the present study the probed deformation region could be more localized, which might also give rise to the apparent discrepancies between the mechanical parameters obtained from different experimental set-ups and operation modes.

Within the dominant deformation mechanism in the context of dislocation, the multiple “pop-ins” (indicated by the arrows in Figure 1a) can be regarded as the trigger of sudden collective activities of dislocation [29,30,31] (such as dislocation generation or movement bursts), giving rise to the seemingly discontinuous plastic deformation during nanoindentation. Such massive dislocation activities are also consistent with the conjectures of the resultant “noisy” features seen in the depth-dependent curves of hardness and Young’s modulus (Figure 1b,c), as well as those reported by Almeida et al. [32] and Jian et al. [22]. The multiple “pop-in” behaviors had also been observed in Reference [32]. From Figure 1a, the first “pop-in” is observed on the loading curve at a load of about 0.08 mN in the present work, which is substantially smaller than that (~0.2 mN) reported previously by Almeida et al. [32]. It is noted that the indenter used in Reference [32] was a cono-spherical-type tip with a radius of ~260 nm equipped in a Hysitron Triboscope nanoindenter system. It is possible that different operating modes, indenter geometrical shapes, and size of radius may lead to the vastly dissimilar nanoindentation results.

However, as mentioned above, when a spherical indenter with a larger tip radius was used, only a single “pop-in” event was observed [21]. This discrepancy, as we discussed above, might originate primarily from the differences in operating modes and the geometric shape of the indenter being used to probe the nanoindentation properties of the same material. The other feature to be noted is that no evidence of reverse discontinuities in the unloading segment (the so-called “pop-out”) can be identified in the present case. This indicates that the pressure-induced phase transformation commonly observed in single-crystal silicon [33] is probably not happening in InP, although the zincblende crystalline structure of InP is in fact not that different from the diamond structure of Si. In any case, in order to gain a more comprehensive understanding of the underlying indentation-induced deformation mechanism, direct microstructural investigations such as SEM and XTEM analyses are certainly indispensable.

### 3.2. XTEM and SAD Analyses

**Figure 3:**
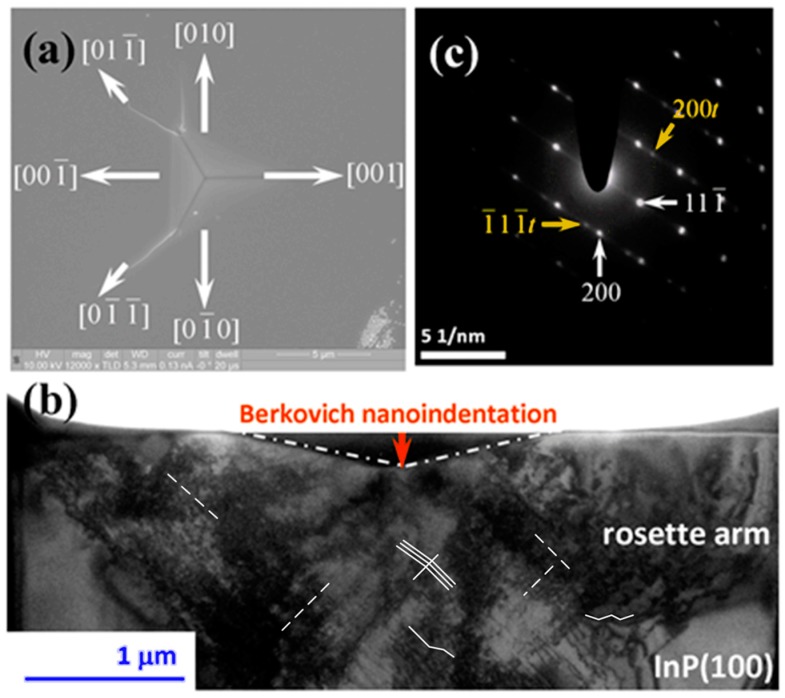
An indented InP(100) single crystal under an indentation load of 150 mN. (**a**) SEM micrograph showing the cracking behaviors. (**b**) Bright-field XTEM image: micro-twins are indicated by solid white lines; dash lines are used to guide the eyes for lattice fringes. (**c**) SAD pattern of sample underneath the Berkovich indenter.

Figure 3a shows a SEM image of the InP(100) single-crystal surface after being indented with a load of 150 mN, featuring the characteristics of Berkovich nanoindentation-induced cracks. Although it appears that the paths of cracking propagation are not straight and the propagation directions are somewhat random, the directions, nevertheless, can be roughly divided into <100> and <110> directions [22]. A bright-field XTEM image of the area immediately beneath the tip of the Berkovich indenter is shown in Figure 3b. It is clearly evident that the slip bands are oriented at ~54.7° to the (100) surface, indicating that the dislocations have been gliding along the <110>{111} slip systems expected for the zincblende-structured InP. The rosette arm patterns ubiquitously observed in materials with similar crystal structure [32,34] are also evident, as indicated in Figure 3b. A closer examination by select area diffraction (SAD) further reveals that the deformation zone immediately beneath the indent consists of a mixture of slip-bands and micro-twins. This is clearly illustrated by the double spots and streaks seen in the [011] zone-axis SAD pattern displayed in Figure 3c. The SAD pattern also indicates that the dislocations and twins are lying parallel on the {111} planes. The direct microstructural observations by XTEM, thus, clearly confirm that indentation-induced deformation in single-crystal InP(100) is exclusively dominated by dislocation activities, and mechanisms such as phase transformation and amorphization are not involved in the nanoindentation process. In the present study, the Berkovich nanoindentation-induced multiple “pop-in” behaviors exhibited on the loading curve are attributed to the formation of slip-bands and micro-twins in single-crystal InP(100). Whereas, based on their observations, Almeida et al. [32] proposed that the multiple “pop-in” behaviors resulted from the Lomer-Cottrell locks, work-hardened region, and a high density of dislocation loops formed beneath the cono-spherical tip. From this viewpoint, the geometrical shape of the indenter tip may have played an important role on the activation of the slip system or/and the formation of dislocations. This may also explain the different number of pop-in events often observed in materials with the same crystal structure during nanoindentation, such as zincblende-structured InP [21,22,32] and hexagonal-structured GaN thin films [14,16].

Moreover, from Figure 3b, the number of dislocations (*N*) generated by the indenter can be roughly estimated using the following expression: *N* = *h*_r_/*b*_z_, where *h*_r_ is the residual depth of the indenter and *b*_z_ is the component of the dislocations along the loading axis [35]. Taking *h*_r_ ~1200 nm, it is estimated that there were about 3000 dislocations formed underneath the indenter tip in this work. This number is about an order of magnitude smaller than the dislocation loops with critical size generated during first pop-ins obtained using thermodynamics energetic estimation (see below). However, considering that the above estimation was made based on the TEM image taken after the entire indentation was completed and the applied stress was removed, this, in fact, is quite consistent. This is because with further increasing load, the dislocation loops formed during the first pop-in may slide and merge to form twins and slip-bands, resulting in multiple pop-ins in the later stage of indentation and reducing the number of dislocations in the residual indentation depth, as revealed in the TEM image. Further, the average dislocation densities (*ρ*) were estimated using the relationship *ρ* = 2*N*/*Lt* [36], where *L* is the total length of random lines projected on a given area of XTEM image and *t* is the foil thickness. In this experimental result, *ρ* is thus estimated in the order of about 10^14^ m^−2^.

### 3.3. Homogeneous Dislocation Nucleation

In the scenario described above, the first pop-in event appearing in the loading segment naturally reflects the onset of plasticity in single-crystal InP(100) manifested by sudden dislocation nucleation and propagation. In other words, the corresponding loading is likely intimately associated with the critical shear stress (*τ*_max_), and the energy associated with the pop-in depth may directly account for the number of indentation-induced newly nucleated dislocation loops. Following the analytical model by Johnson [37], *τ*_max_ can be related to an indentation load (*P*_c_) at which a discontinuity in the load-displacement curve takes place, through the following equation:

(2) $$\tau_{\max}=0.31{\left(\frac{6P_{c}E_{r}^{2}}{\pi^{3}R^{2}}\right)}^{1/3}$$

where *R* is the radius of the indenter tip. Thus, the obtained *τ*_max_ for single-crystal InP(100) is about 2.3 GPa. We assume that the *τ*_max_ is responsible for the homogeneous dislocation nucleation underneath the indenter tip. Therefore, the shear stress that initiates plastic deformation and the energy required for generating a dislocation loop to prevail the deformation can be estimated from the present data. The free energy (*U*) of a circular dislocation loop of radius (*r*) is given as:

(3) $$U=\gamma_{dis}2\pi r-\tau b\pi r^{2}$$

where $\gamma_{dis}$ is the line energy of the dislocation loop, *b* is the magnitude of Burgers vector (~0.4 nm) [38], and *τ* is the external shear stress acting on the dislocation loop. The energy required to create a dislocation loop in a defect-free lattice is described in the first term on the right-hand side of Equation (3) and is also equal to the increased lattice energy due to the formation of a dislocation loop. The second term of Equation (3) is nothing but the strain energy released via work done by the applied stress (*τ*) to expand the dislocation loop over a displacement of one Burgers vector. The lattice strain in the vicinity of the dislocation for *r* > *r_core_*, thus $\gamma_{dis}$, is given by Reference [25]:

(4) $$\gamma_{dis}=\frac{Gb^{2}}{8\pi }\frac{2-v_{InP}}{1-v_{InP}}\left[\ln\left(\frac{4r}{r_{core}}\right)-2\right]$$

where *G* is the shear modulus, and for single-crystal InP(100) *G* ≈ 31 GPa [39]. The value of the radius of dislocation core *r_core_* is usually assumed to be about one lattice constant. By using Equations (1), (3) and (4) can be rewritten as:

(5) $$U=\frac{Gb^{2}}{4}\left(\frac{2-v_{InP}}{1-v_{InP}}\right)\left(\ln\frac{4r}{r_{core}}-2\right)r-\pi br^{2}\tau_{c}$$

This relates the material properties and observed pop-in load to the free energy responsible for dislocation nucleation. The resolved shear stress (*τ*_c_) is usually taken as the half of *τ*_max_ [39]. The *U* has a maximum at a critical radius (*r*_c_) above which the system gains energy by increasing *r*. According to Equation (5), this maximum energy decreases with increasing load and a pop-in, i.e., the homogeneous formation of a circular dislocation loop becomes possible without thermal energy at *U* = 0 [40]. With this condition and setting d*U*/d*r* = 0 for a maximum, this yields $\tau_{c}=2\gamma_{dis}/br$ and $r_{c}=\left(e^{3}r_{core}\right)/4$. Consequently, *r_core_* ≈ 0.43 nm and *r_c_* = 2.15 nm are obtained. The value *r_core_* ≈ 0.43 nm is slightly smaller than *a* ≈ 0.587 nm for InP, however, it is consistent with the atomic distance along the <110> orientation (≈0.42 nm), indicating that the above analysis is reasonable. The fact that the critical radius of the dislocation loop *r_c_* (=2.15 nm) needs to extend over a distance of about 5 times the dislocation core to become stable is also interesting. This suggests that the system is more prone to accommodate strain energy with larger dislocations loops.

**Figure 4:**
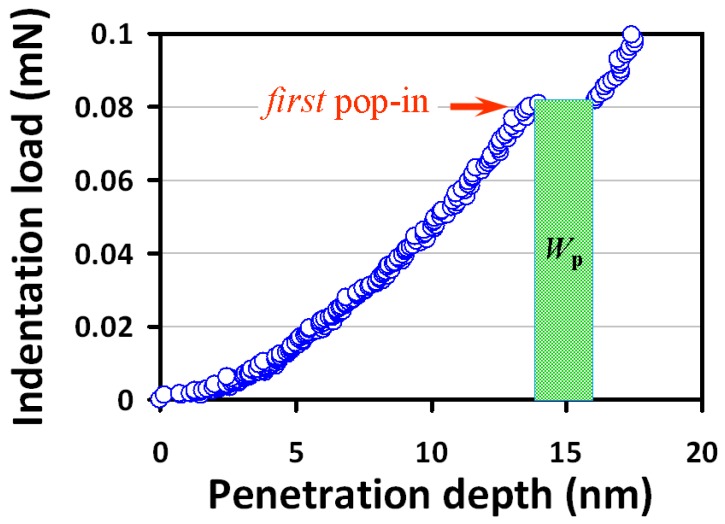
The corresponding pop-in event (red arrow) from Figure 2a is zoomed in. The plastic strain work is *W*_p_: critical loading × sudden incremental displacement.

The number of dislocation loops formed in the first pop-in can be estimated from the associated work (*W*_p_) done during nanoindentation. As depicted in Figure 4, *W*_p_ is estimated to be about 0.2 × 10^−12^ Nm, implying that ~3 × 10^4^ dislocation loops with a radius larger than *r_c_* have been generated during the pop-in event. This number is relatively low and consistent with the scenario of homogeneous dislocation nucleation-induced pop-in, instead of activated collective motion of pre-existing dislocations [15]. Moreover, as discussed above, this number is also in line with the number of dislocations estimated from the TEM image taken from the region of residual indentation depth. Alternatively, one can take the total dissipation energy as the energy to estimate the number of dislocations with critical radius being generated during entire nanoindentation practice. In that case, as many as ~10^6^ dislocation loops may have been formed during nanoindentation. However, this number can only be regarded as an upper limit because it is quite unlikely that all the dissipated indentation energy is completely transferred to form dislocation loops within the deformation region in single-crystal InP(100).

### 3.4. Vickers Indentation Induced Fracture Behavior

**Figure 5:**
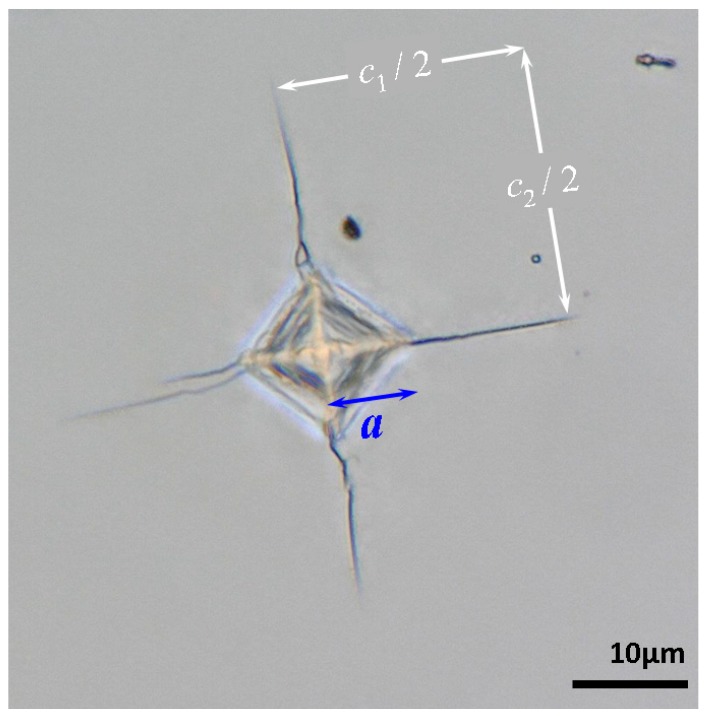
Vickers indentation at 1.96 N in single-crystal InP(100): Palmqvist cracks emitting from Vickers indentation, where *a* is the half-diagonal of the indentation and *l* = (*c*_1_ + *c*_2_)/4 is the average length of the radial cracks for each indentation.

Fracture toughness (*K_C_*) can be readily measured by the induced cracks at the corners of indentations made on the substrate materials. This method is known as the indentation microfracture [41,42,43]. Figure 5 shows the Vickers-indentation-induced cracking pattern on single-crystal InP(100) at a loading of 1.96 N. It can be found that the ratio of average cracking length (*l* = (*c*_1_ + *c*_2_)/4, where *c*_1_ and *c*_2_ are defined in Figure 5) to the half-diagonal of the indentation (*a*) meets the criteria of Palmqvist cracks with 0.25 ≤ *l*/*a* ≤ 2.5. Therefore, the formula proposed by Niihara et al. [41,42] was adopted to calculate the *K_C_* of single-crystal InP(100) here, as follows:

(6) $$K_{C}=0.009{\left(\frac{E_{InP}}{H}\right)}^{2/5}\;\frac{P_{a}}{a\sqrt{l}}$$

where *P_a_* is the applied load. The *K_C_* of single-crystal InP(100) obtained is about 1.2 MPa·m^1/2^. Comparing to the *K*_IC_ values of 0.42~0.53 MPa·m^1/2^ for InP reported by Ericson et al. [44], the present result seems slightly too large. However, considering that *K*_C_ is strongly dependent on specimen geometry and usually decreases with increasing thickness to reach a minimum value known as *K*_IC_, we believe that a factor of 2–2.5 difference is quite reasonable. Finally, the fracture energy (*G_C_*) can also be calculated using the equation $G_{C}=K_{C}^{2}\left[\left(1-v_{InP}^{2}\right)/E_{InP}\right]$ [45], yielding approximately 14.1 J·m^−2^ for single-crystal InP(100). This number indicates that InP is, in fact, quite ductile, which is also consistent with the dislocation-dominated deformation discussed above.

## 4. Conclusions

To sum up, the nano- and micro-scale deformation mechanisms and behaviors of single-crystal InP(100) were studied by combining indentation, SEM, and XTEM techniques. From the XTEM results and SAD analysis, the nanoindentation responses of single-crystal InP(100) involve the the formation of slip-bands and micro-twins. Preliminary energetic estimations indicate that the number of dislocation loops induced by nanoindentation to trigger the plastic deformation accounting for the first pop-in event was in the order of 10^4^ with a critical radius *r_c_* ≈ 2.15 nm. Furthermore, from Vickers indentation tests, the obtained values of *K_C_* and *G_C_* of single-crystal InP(100) were about 1.2 MPa·m^1/2^ and 14.1 Jm^−2^, respectively.
